# Changes in the prevalence of intellectual disability among 10-year-old children in Sweden during 2011 through 2021: a total population study

**DOI:** 10.1186/s11689-024-09576-3

**Published:** 2024-10-23

**Authors:** Maki Morinaga, Viktor H. Ahlqvist, Michael Lundberg, Anna-Clara Hollander, Dheeraj Rai, Cecilia Magnusson

**Affiliations:** 1 Department of Global Public Health, Karolinska Institutet, Stockholm, 171 77 Sweden; 2 Centre for Academic Mental Health, Population Health Sciences, Bristol Medical School, University of Bristol, Bristol, BS8 1QU UK; 3 NIHR Biomedical Research Centre, University of Bristol, Bristol, BS8 2BN UK; 4 Avon and Wiltshire Partnership National Health Service Mental Health Trust, Bath, BA1 3QE UK

**Keywords:** Intellectual disability, Autism spectrum disorder, Prevalence, Epidemiology, Sociodemographic factors, Low birth weight, Prematurity

## Abstract

**Background:**

Recent studies have suggested an increasing prevalence of intellectual disability diagnoses in some countries. Our aim was to describe the trend in the prevalence of intellectual disability diagnoses in Sweden and explore whether associated sociodemographic and perinatal factors can explain changes in the prevalence.

**Methods:**

We used a register-based nationwide cohort of residents in Sweden born between 2001 and 2011. We calculated the prevalence of intellectual disability diagnoses by age 10 for each birth cohort and the prevalence ratios in relation to the baseline year 2011, overall and by severity of intellectual disability, and comorbidity of autism and attention-deficit/hyperactivity disorder. The prevalence ratios were stratified and adjusted for associated sociodemographic and perinatal factors.

**Results:**

Among 1,096,800 individuals, 8,577 were diagnosed with intellectual disability by age 10. Among these, 3,949 (46%) and 2,768 (32%) were also diagnosed with autism and attention-deficit/hyperactivity disorder, respectively, and 4% were diagnosed with profound, 8% severe, 20% moderate, 52% mild, and 16% other/unspecific intellectual disability. The recorded age-10 prevalence of intellectual disability diagnoses increased from 0.64% (95% confidence interval 0.59–0.69%) in 2011 to 1.00% (0.94–1.06%) in 2021, corresponding to an annual prevalence ratio of 1.04 (1.04–1.05). The increase was, however, restricted to mild, moderate, and other/unspecific intellectual disability diagnoses, while the trends for profound and severe intellectual disability diagnoses were stable. The increasing trend was perhaps less pronounced among females and children with diagnosed attention-deficit/hyperactivity disorder, but independent of the co-occurrence of autism. The prevalence ratios did not change with stratification or adjustment for other associated demographic and perinatal factors.

**Conclusion:**

The recorded prevalence of diagnosed mild and moderate intellectual disability among 10-year-olds in Sweden has increased over the recent decade. This increase could not be explained by changes in associated sociodemographic or perinatal factors, including birth weight, gestational age, and parental age, migration status, and education at the child’s birth. The increase instead may be due to changes in diagnostic practices in Sweden over time.

**Supplementary Information:**

The online version contains supplementary material available at 10.1186/s11689-024-09576-3.

## Background

Intellectual disability (ID) is characterized by both intellectual and adaptive functioning deficits in conceptual, social, and practical skills with the onset during the developmental period (typically childhood and adolescence) [1]. ID often severely impacts the health, quality of life, and welfare of individuals and their families. According to the Diagnostic and Statistical Manual of Mental Disorders Fifth Edition (DSM-5), the severity of ID is classified by the level of support required; mild if a person can live independently with minimal levels of support, moderate if a person can live independently with moderate levels of support, severe if a person needs daily assistance for self-care activities and safety supervision, and profound if a person needs 24-hour care [1]. Globally, ID ranks as the third leading cause of Disability-Adjusted Life Years for mental disorders among children aged 0 to 14 years and the seventh leading cause among all ages [2].

The current prevalence of ID diagnoses is estimated at 1.04% globally according to a meta-analysis published in 2011 [3]. Nonetheless, the information on global time trends in prevalence of ID diagnoses is scarce, despite the substantial increases observed in other neurodevelopmental disorders such as autism and attention-deficit/hyperactivity disorder (ADHD) [4–6]. A few existing recent studies from the USA, Finland, and Australia have reported an increase in prevalence of ID diagnoses over the last decade [7–10]. The Australian study concluded that most of the increase concerned mild or moderate ID, and may be partly attributable to an increase in prevalence of autism diagnoses [9]. Yet, studies on the time trend of prevalence of ID diagnoses are lacking in most countries, including Sweden. These studies are needed for better planning of health, education, and social services.

Several sociodemographic and perinatal factors have been associated with ID, including male sex, advanced parental age, preterm birth, low birth weight [11], lower parental education [12], and parental migration status [13]. Changes in the distribution and occurrence of such associated factors over time may have contributed to a change in the prevalence of ID. For instance, parental reproductive age has increased both globally and in Sweden in recent decades. Improvements in the care of extremely preterm infants have also led to a higher survival rate for very and extremely preterm births in Sweden [14]. Additionally, the number of international migrants has drastically increased, reaching 272 million worldwide in 2019 [15], and comprising 17.6% of the Swedish population [16]. However, it remains unclear whether such changes in sociodemographic and perinatal factors over time have contributed to changes in the prevalence of ID diagnoses.

Moreover, some factors are also associated with the identification of ID. For instance, the age at which ID is first recorded has been reported to peak at different ages between sexes, such as age 5 for boys and age 14 for girls [17]. In addition, lower parental education and parental migration status could impact the identification of ID cases by influencing healthcare utilization and being associated with disparities in ascertainment or referral patterns [18, 19]. These patterns and disparities, and therewith identification of ID within such population groups, may also have changed over time, contributing to changing prevalences of ID diagnoses.

In this study, we examined how the recorded prevalence of ID diagnoses has changed over time in Sweden and investigated whether these changes can be explained by concomitant shifts in sociodemographic and perinatal factors associated with the identification or occurrence of ID.

## Methods

### Study Population

We used a nationwide total population cohort in Sweden with prospectively recorded information through record linkage with a range of Swedish health and administrative registers. The record linkage was accomplished using the unique personal identification number assigned to each resident at birth or upon arrival in Sweden for migrants. Our study population included all individuals born between January 1, 2001, and December 31, 2011, who resided in Sweden at any time during the follow-up period until December 31, 2021. We excluded children who resided for less than 4 years in Sweden to ensure an adequate follow-up time for children to receive a diagnosis of the outcome. The study was approved by the Swedish Ethical Review Authority (DNR 2020–05516, 2021-05958-02, and 2022-05648-02).

### Case assessment

The National Patient Register (NPR) encompasses nationwide data on all inpatient care with complete national coverage since 1987 and outpatient specialist care since 2001. We identified our primary outcome, ID by 10 years of age, according to the first day of registered International Classification of Diseases (ICD)-9 (317–319) and ICD-10 (F70-F79) codes in the NPR. Among children with an ID diagnosis by age 10, those who ever received a diagnosis of autism or ADHD based on ICD-9 (299 for autism and 314 for ADHD) and ICD-10 (F84 for autism and F90 for ADHD) until the end of 2021 were classified as ID with autism and ID with ADHD, respectively. The severity of ID was categorized as profound, severe, moderate, mild, and other/unspecific based on the ICD codes. For children with multiple ID diagnoses of varying severity (31% of all children diagnosed with ID), the most severe diagnosis was retained. This decision was made because the majority (81%) of these children had other/unspecified ID in addition to their specified diagnosis.

### Covariates

Information on the child’s date of birth, sex, and other perinatal factors, and first-degree biological relatives and their date of birth, birthplace, and education was identified from the Medical Birth Register, the Multi-generation Register, the Register of Total Population, and the longitudinal integrated database for health insurance and labor market studies (LISA) [20]. Parental education at the child’s birth was categorized as < 10, 10–12, ≥ 13 years. Parental migration status was categorized based on parental country of birth: children with both parents born in Sweden, both parents born abroad, mother born abroad and father born in Sweden, father born abroad and mother born in Sweden. Maternal age at the child’s birth was classified as < 25, 25–29, 30–34, ≥ 35 years old, and paternal age at the child’s birth as < 25, 25–29, 30–34, 35–39, ≥ 40 years old. Birth weight was categorized as: extremely low < 1000 g, very low 1000–1499 g, low 1500–2499 g, normal 2500–4499 g, high ≥ 4500 g. Gestational age was classified as: extremely preterm < 28 weeks, very preterm 28–31 weeks, moderate to late preterm 32–36 weeks, term 37–41 weeks, post-term ≥ 42 weeks.

### Statistical analysis

We initially calculated the cumulative prevalence of ID by age 10 for each birth cohort. We then estimated the ratios of the prevalence of diagnosis by age 10 for each year during the follow-up, compared to the reference year of 2011, and the average relative increase per year across the entire study period. These prevalence and prevalence ratios and their 95% confidence intervals were estimated using generalized estimating equations log-binomial models [21]. To examine whether changes in the prevalence of ID can be explained by sociodemographic and perinatal factors associated with the occurrence of ID, i.e. birth weight, gestational age, and parental migration status, age, and education at the child’s birth, we adjusted the models for such factors and if the prevalence ratios remained statistically significant (*p* < 0.05) after adjustment, we concluded that these factors do not explain the trend seen in the crude analysis. We repeated the calculations according to co-occurrence of autism and ADHD, as well as according to the severity of ID. Additionally, we calculated the cumulative prevalence and the prevalence ratios stratified by sociodemographic factors, including sex and parental migration status and education at the child’s birth, and conducted Wald tests to calculate the p-value for the interaction (product-term) between calendar year and the sociodemographic factors. Moreover, as a sensitivity analysis, we repeated the calculations using a cohort including both individuals born in Sweden and abroad (*n* = 1,200,450), with adjustment for only parental age and migration status at the child’s birth, because the exclusion of the individuals without data on perinatal factors in the main analysis led to excluding all children born abroad. In addition, we repeated the calculations using a categorization of severity based on the last diagnosis if a person had multiple registered diagnoses. All analyses were conducted in SAS version 9.4.

## Results

In the total study population of 1,381,285 individuals, we excluded children who resided for less than 4 years in Sweden during the follow-up period (*n* = 69,033), children without data on perinatal factors such as birth weight and gestational age (*n* = 205,008), and children without parental data on age, education, and migration status (*n* = 10,444), leaving 1,096,800 individuals for analysis. We identified a total of 8,577 individuals who received an ID diagnosis before or at age 10, of whom 3,949 (46%) and 2,768 (32%) had an autism and ADHD diagnosis, respectively. Among those with ID diagnosis, 311 (4%) were diagnosed with profound, 670 (8%) with severe, 1,700 (20%) with moderate, 4,488 (52%) with mild, and 1,408 (16%) with other/unspecific ID. A larger proportion of mild cases was recorded among children with diagnosed ID without autism (57% compared with 47% among those with diagnosed ID with autism), but it was opposite for ADHD (47% among children with diagnosed ID without ADHD compared with 64% among those with ID with ADHD).

Some changes in sociodemographic and perinatal factors over time were observed (Supplementary Table 1). Parental ages and education at the child’s birth increased from 2001 to 2011. Specifically, the share of children with high maternal (≥ 35 years) and paternal age (≥ 40 years) at the child’s birth increased (from 21% to 25% and 13% to 17%, respectively), while those with maternal age 25–29 years and paternal age 25–34 years decreased. Children with parental education ≥ 13 years also increased (from 49% to 60%), while those with parental education 10–12 years decreased (from 47% to 35%). Additionally, children with a parental migration background, especially those with both parents born abroad increased (from 12% to 17%), while those with both parents born in Sweden decreased (from 77% to 70%). On the other hand, the distributions of gestational age and birth weight did not change materially over time, except for minimal decreases in children with gestational age ≥ 42 weeks and those with birth weight ≥ 4500 grams.

**Fig. 1:**
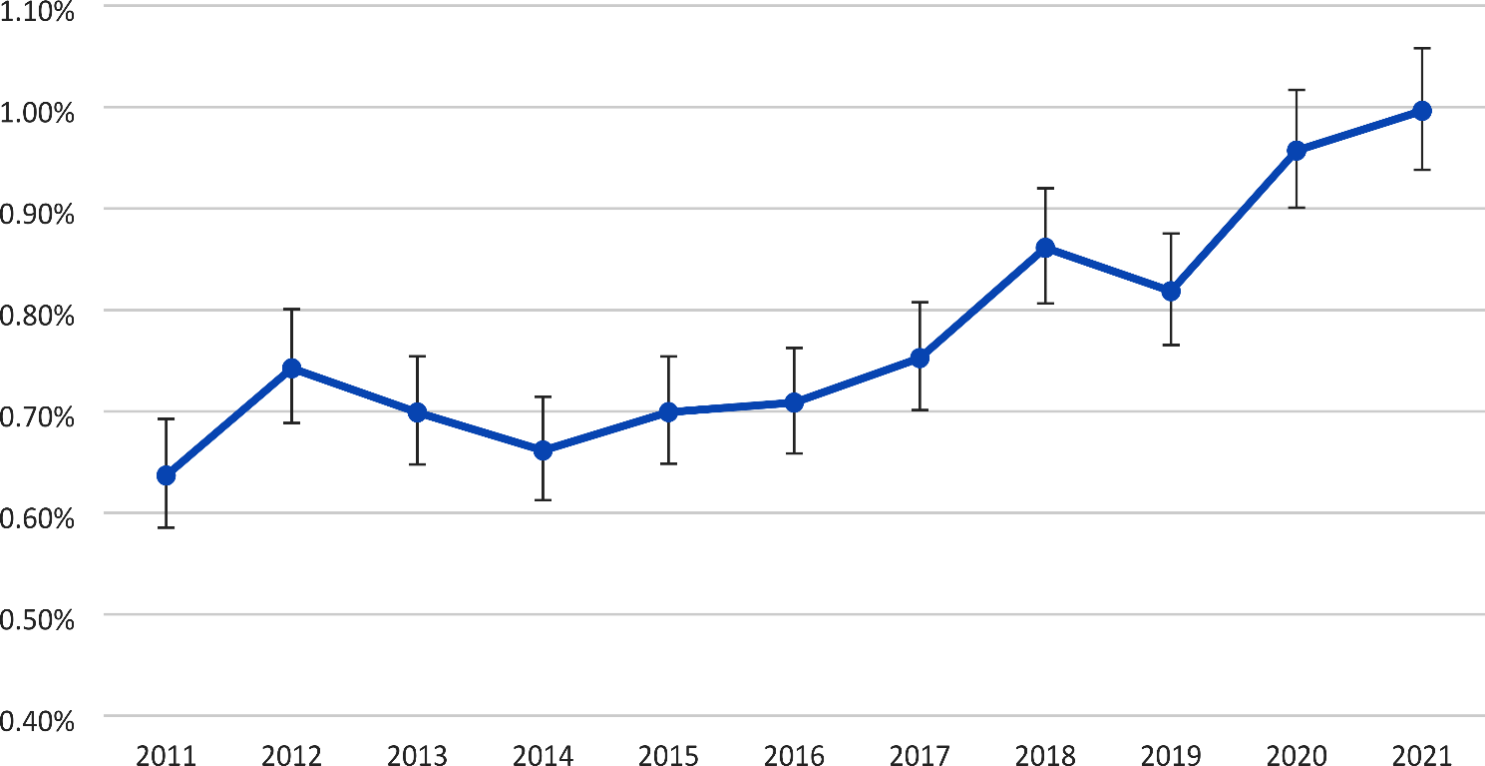
Prevalence of intellectual disability among children aged 10 years by calendar year (%)

**Fig. 2:**
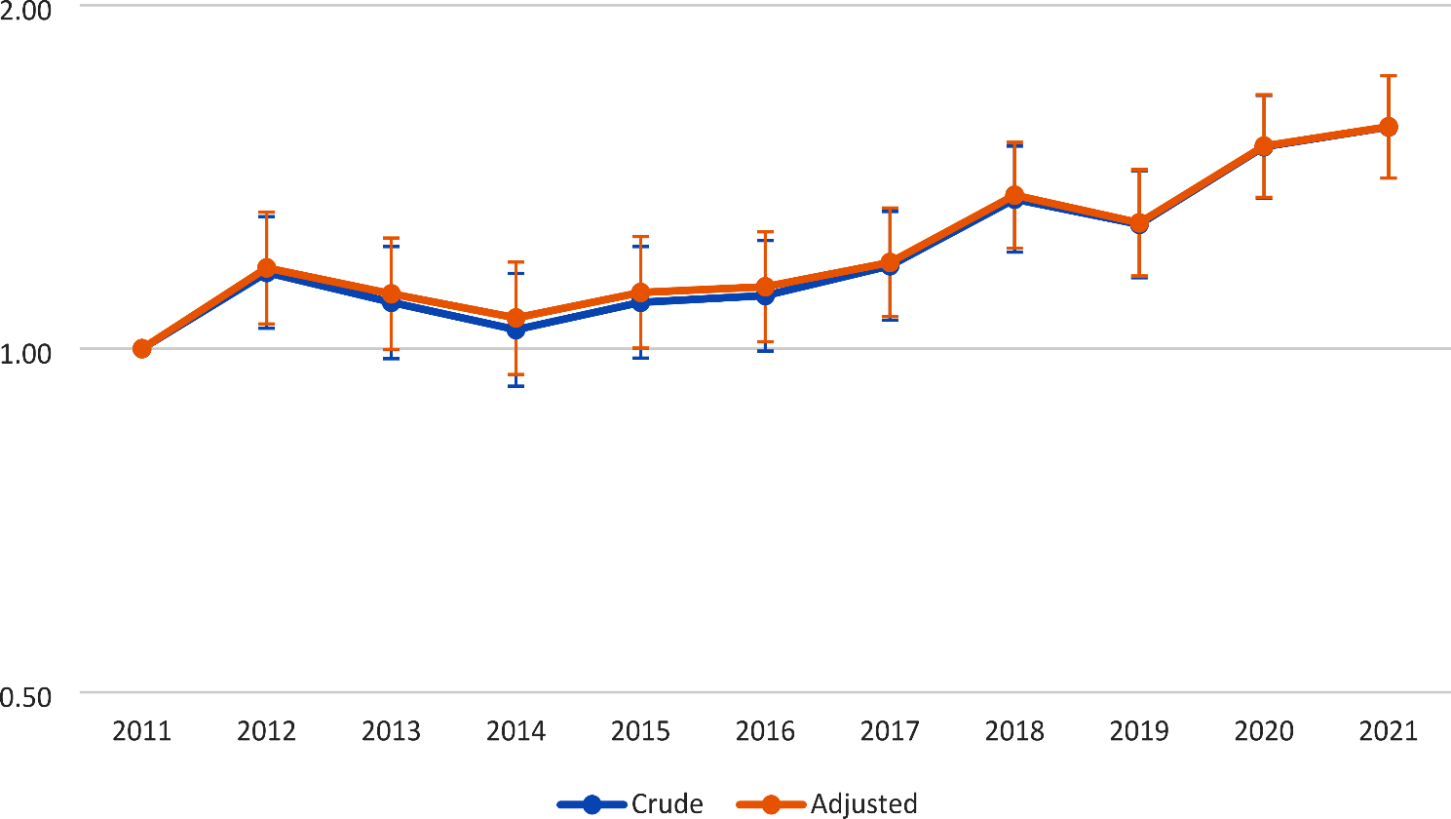
Changes in intellectual disability among children aged 10 years depicted as crude and adjusted ^a^prevalence ratios in relation to the baseline year 2011. **a**. Adjusted for birth weight, gestational age, and parental migration status, age, and education at the child’s birth

The age-10 prevalence of ID diagnosis was 0.64% (95% CI 0.59–0.69%) in 2011, and increased with calendar years, reaching 1.00% (0.94–1.06%) in 2021 (Fig. 1). This translates into a crude prevalence ratio of 1.56 (1.41–1.74) comparing 2021 to 2011 (Fig. 2). On average, the prevalence in 10-year-olds increased by 4% per year relative to the previous year (the average prevalence ratio of 1.04 (1.04–1.05)).

**Fig. 3:**
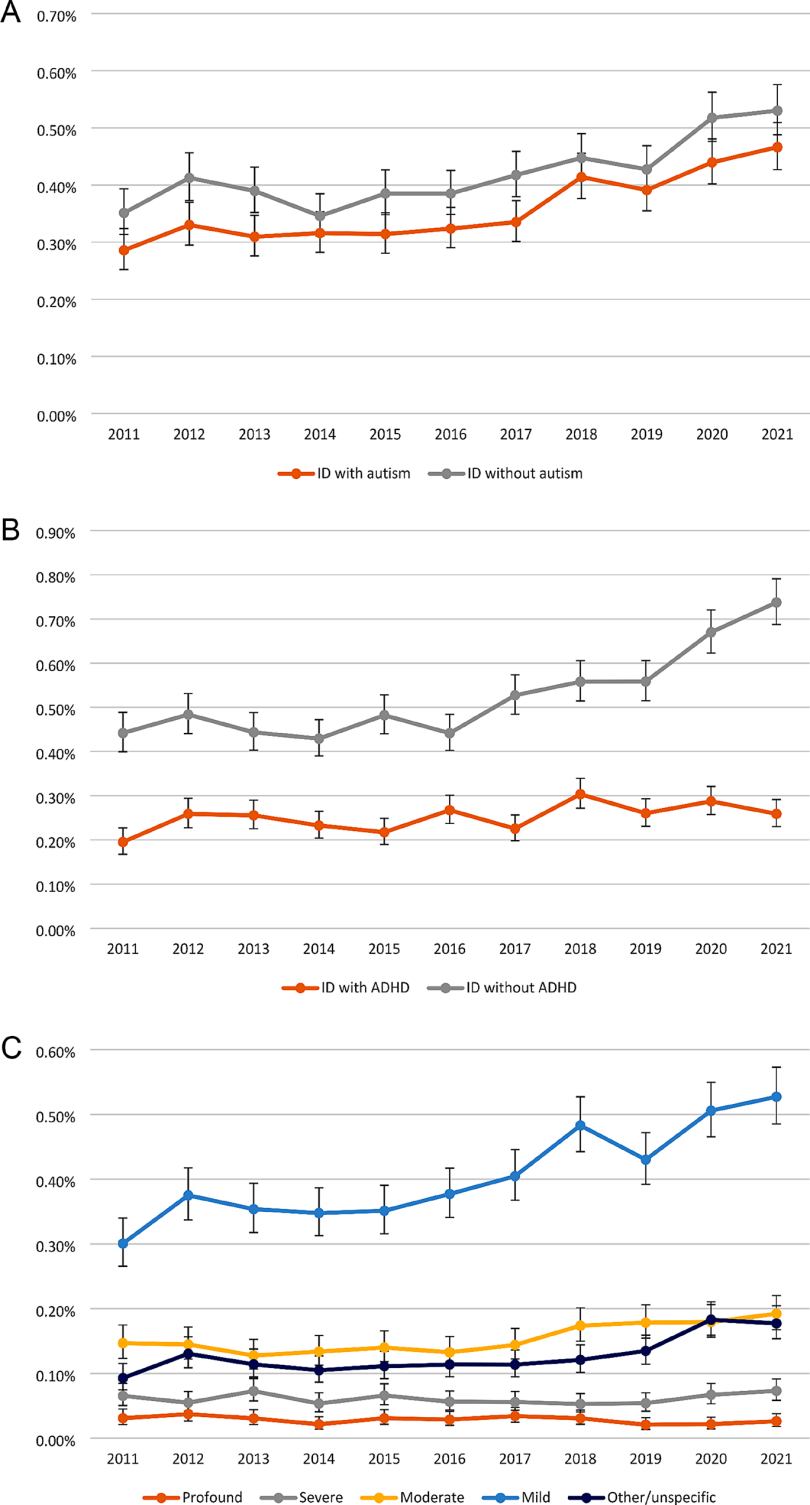
Prevalence of intellectual disability among children aged 10 years by co-occurrence of autism and ADHD and severity of ID (%). ID = intellectual disability; ADHD = attention-deficit/hyperactivity disorder. **a**. Co-occurrence of autism. **b**. Co-occurrence of ADHD. **c**. Severity of ID

Similar trends were observed regardless of comorbid autism (Fig. 3a and Supplementary Fig. 1), with increased age-10 prevalence from 2011 to 2021. The prevalence for ID with autism diagnosis rose from 0.29% (0.25–0.32%) to 0.47% (0.43–0.51%) with an average prevalence ratio of 1.05 (1.04–1.06). For ID without autism diagnosis, the prevalence increased from 0.35% (0.31–0.39%) to 0.53% (0.49–0.58%) with an average prevalence ratio of 1.04 (1.03–1.05). However, differences were noted in the trend of the age-10 prevalence by co-occurrence of ADHD (Fig. 3b and Supplementary Fig. 1). The prevalence of ID without ADHD increased from 0.44% (0.40–0.49%) to 0.56% (0.51–0.61%) with an annual prevalence ratio of 1.05 (1.04–1.06). In contrast, the prevalence of ID with ADHD increased only from 0.20% (0.17–0.23%) to 0.26% (0.23–0.29%) with an average prevalence ratio of 1.02 (1.01–1.03).

The age-10 prevalence trends for ID also varied according to the severity of the ID diagnosis (Fig. 3c and Supplementary Fig. 2). The prevalence of other/unspecified ID increased from 0.09% (0.07–0.12%) to 0.18% (0.15–0.20%) with an average prevalence ratio of 1.06 (1.04–1.07), mild ID increased from 0.30% (0.27–0.34%) to 0.53% (0.49–0.57%) with an average prevalence ratio of 1.05 (1.04–1.06), and moderate ID rose from 0.15% (0.12–0.17%) to 0.19% (0.17–0.22%) with an average prevalence ratio of 1.04 (1.02–1.05). Severe and profound ID diagnoses instead remained stable during the period, with an average prevalence ratio of 1.00 (0.98–1.03) for severe and 0.97 (0.94–1.01) for profound ID. The sensitivity analysis using a categorization of severity based on the last diagnosis showed higher prevalence of other/unspecific ID, but otherwise generally similar trends in each diagnostic class over time (Supplementary Fig. 3).

The prevalence ratios for overall ID diagnosis and all subgroups of ID diagnosis barely changed after adjusting for birth weight, gestational age, and parental migration status, age, and education at the child’s birth (Fig. 2 and Supplementary Table 2 for overall ID and Supplementary Figs. 1, 2 for subgroups of ID). There were only small changes after the adjustment of all covariates at the same time or each covariate at a time, with some variations by calendar year. For instance, the adjusted prevalence ratios of overall ID diagnosis changed by between 0.06% in 2021 and 2.41% in 2014 compared with the crude ones.

The time trends of the age-10 prevalence were similar across strata defined by parental migration status and education at the child’s birth (p-values ranging from 0.83 to 0.88 for the interaction term between calendar year and the respective sociodemographic factor) (Table 1). In contrast, the prevalence increased slightly more among males than among females during the last years of the study period (a p-value < 0.05 for the interaction term).

**Table 1:** Changes in intellectual disability among children aged 10 years depicted as prevalence ratios in relation to the baseline year 2011, stratified by sociodemographic factors

Calendar year	Main analysis	Sex		Parental education at birth			Parental migration status			
		Female	Male	< 10 years	10–12 years	≥ 13 years	Both native-born parents	Both foreign-born parents	Foreign-born mother and native-born father	Native-born mother and foreign-born father
2011	Ref.	Ref.	Ref.	Ref.	Ref.	Ref.	Ref.	Ref.	Ref.	Ref.
2012	1.17(1.04–1.31)	1.16 (0.97–1.39)	1.17 (1.01–1.35)	1.26 (0.86–1.83)	1.19 (1.02–1.38)	1.15 (0.95–1.39)	1.18 (1.03–1.35)	1.22 (0.94–1.58)	1.04 (0.64–1.67)	1.01 (0.66–1.57)
2013	1.10 (0.98–1.23)	1.06 (0.88–1.27)	1.12 (0.97–1.30)	1.34 (0.93–1.94)	1.11 (0.95–1.29)	1.10 (0.91–1.33)	1.06 (0.92–1.22)	1.40 (1.09–1.79)	0.80 (0.49–1.32)	0.90 (0.58–1.39)
2014	1.04 (0.93–1.16)	1.03 (0.86–1.24)	1.04 (0.90–1.21)	1.35 (0.93–1.95)	1.06 (0.90–1.23)	1.05 (0.87–1.26)	0.99 (0.86–1.14)	1.27 (0.99–1.63)	1.07 (0.68–1.70)	0.76 (0.48–1.20)
2015	1.10 (0.98–1.23)	0.96 (0.80–1.16)	1.19 (1.03–1.37)	1.27 (0.88–1.83)	1.14 (0.97–1.32)	1.12 (0.93–1.35)	1.04 (0.90–1.19)	1.28 (1.00-1.64)	0.95 (0.59–1.51)	1.13 (0.75–1.71)
2016	1.11 (1.00-1.24)	1.05 (0.88–1.26)	1.16 (1.00-1.33)	1.45 (1.01–2.07)	1.14 (0.98–1.33)	1.13 (0.94–1.36)	1.01 (0.88–1.16)	1.36 (1.07–1.73)	1.06 (0.68–1.66)	1.17 (0.78–1.77)
2017	1.18 (1.06–1.32)	1.23 (1.03–1.46)	1.15 (1.00-1.33)	1.38 (0.97–1.97)	1.25 (1.07–1.45)	1.20 (1.00-1.44)	1.12 (0.98–1.28)	1.38 (1.09–1.74)	0.91 (0.57–1.45)	1.05 (0.70–1.59)
2018	1.35 (1.22–1.51)	1.22 (1.03–1.46)	1.44 (1.25–1.64)	1.72 (1.22–2.41)	1.38 (1.19–1.60)	1.41 (1.18–1.68)	1.30 (1.14–1.48)	1.45 (1.15–1.83)	1.04 (0.66–1.63)	1.31 (0.88–1.95)
2019	1.29 (1.15–1.43)	1.12 (0.94–1.33)	1.40 (1.22–1.60)	1.56 (1.11–2.20)	1.44 (1.24–1.67)	1.23 (1.03–1.47)	1.18 (1.03–1.35)	1.43 (1.14–1.81)	1.27 (0.82–1.96)	1.19 (0.80–1.77)
2020	1.50 (1.36–1.67)	1.31 (1.10–1.55)	1.63 (1.43–1.86)	2.00 (1.44–2.77)	1.46 (1.26–1.69)	1.63 (1.38–1.93)	1.41 (1.24–1.60)	1.70 (1.36–2.12)	1.22 (0.79–1.87)	1.18 (0.79–1.76)
2021	1.56 (1.41–1.74)	1.42 (1.20–1.68)	1.67 (1.46–1.90)	1.79 (1.28–2.49)	1.61 (1.39–1.86)	1.68 (1.42–1.99)	1.48 (1.30–1.69)	1.64 (1.31–2.05)	1.34 (0.88–2.05)	1.34 (0.91–1.99)
2011-2021^a^	1.04 (1.04–1.05)	1.03 (1.02–1.04)	1.05 (1.04–1.06)	1.06 (1.03–1.08)	1.04 (1.03–1.05)	1.05 (1.04–1.06)	1.04 (1.03–1.05)	1.04 (1.02–1.05)	1.04 (1.00-1.07)	1.04 (1.01–1.06)

a. Average annual relative increase from 2011 to 2021

## Discussion

We found that the prevalence of ID diagnoses recorded by health services by age 10 increased between 2011 and 2021 in Sweden, especially in later years. The age-10 prevalence of mild, moderate, and other/unspecific ID diagnoses increased between 2011 and 2021, while the prevalence of profound and severe ID diagnoses was more stable. The increasing trend was perhaps less pronounced among females and children with diagnosed attention-deficit/hyperactivity disorder, but independent of co-occurrence of autism. The trend did not change after adjustment or stratification of birth weight, gestational age, and parental age, migration status, and education at the child’s birth.

Our findings showing an increase of the age-10 prevalence of ID diagnoses in later years align with the previous studies in the USA, Australia, and Finland [7–10]. However, the underlying mechanism of the increase remains elusive. We could not explain the increasing prevalence by either within-strata changes or population shifts in associated sociodemographic and perinatal factors. Particularly, the occurrences of preterm birth and low birth weight did not change significantly over time during the study period, and thus did not explain the increase of ID diagnoses in our results. Yet, there were some differences by sex, wherein diagnoses among males were increasing somewhat more than females in recent years. Further studies are needed to evaluate this trend.

The increase in ID diagnoses was not limited to cases with co-occurring ADHD or autism. In fact, the opposite was true for ADHD. The increase in the prevalence of ID with ADHD diagnoses was marginal, although ADHD diagnoses has increased in Sweden [22] and the majority of individuals with ID with ADHD diagnoses had been diagnosed with mild ID. Therefore, we speculate that the rise in ID is unlikely to be explained as a secondary finding of the diagnostic processes initiated for children with issues related to ADHD or autism. Our interpretation is supported by the notion that autism without co-occurring ID increased sharply, while the prevalence of autism with ID remained fairly stable in Sweden [5]. However, changes in general awareness of neurodevelopmental conditions and access to health care services and diagnostic testing [23] may still have partly contributed to the increase of the prevalence of ID diagnoses.

Our results showed that the prevalence of mild ID diagnoses has especially been increasing, which is in line with the Australian study [9]. The increase may be due to changes in diagnostic practices in Sweden over time. Firstly, a detection of milder cases can be speculated to be possibly improved over time, which may be attributable to changes in awareness of ID, access to health care services, and service availability. For instance, the capacity of child and adolescent psychiatric care has increased in recent years in Sweden [24]. Secondly, change in the diagnosis criteria in DSM-5 in 2013, removing the intelligence quotient score, might have led to that individuals who previously did not meet the criteria now receiving the diagnosis. Thirdly, in connection with school reforms in Sweden implemented between 2011 and 2014, the requirements regarding placement of pupils in special schools or in special teaching groups became stricter [25]. This change possibly contributed to a need for a diagnosis in receiving required support. Moreover, such changes in diagnostic practices might not only lead to an increased number of diagnoses in individuals who would previously not have received the diagnosis but also affect the child’s age at diagnosis. An individual who would previously have received the diagnosis later in life may now instead receive the diagnosis earlier, which possibly partly explains the increase in the age-10 prevalence of ID diagnoses.

On the other hand, the prevalence of profound and severe ID diagnoses did not change over time, which may be explained by them being diagnosed earlier [26] and with greater presentation, thereby being less affected by such changes in diagnostic detection. In addition, profound and severe ID have been indicated to be etiologically distinct from mild and moderate ID, which are sometimes hypothesized to be caused by the same genetic and environmental influences responsible for the normal distribution of IQ [27]. Profound and severe ID are suggested to be associated with non-inherited genomic change such as de novo point mutations or imprinting [27]. Our results align with such genetic or chromosomal abnormalities not having increased significantly during the study period.

### Strength and limitations

The primary strength of this study lies in the nationwide data coverage with the large total population sample, which made it possible to examine rare categories of the covariates and outcome. However, there are also limitations. Firstly, we may have missed cases of ID because the outcome was obtained using only the NPR. The register of outpatient specialist care started in 2001 in the NPR with subsequent years for complete coverage, which may have led to incorrect ages of first diagnosis, especially in the early years. In addition, individuals who were diagnosed outside of the hospital are not registered in the NPR. However, we believe that the missing number should be small because even children born in 2001 were followed until 2011, by when the register coverage should have improved, and because most children assessed for ID receive such care by a team of clinical experts within hospital departments, outpatient specialist care, or habilitation centers. Secondly, our choice of the age for assessment might have been inadequate. A previous study indicated that cumulative prevalence of ID by year until age 18 would provide a better estimate of the prevalence, although the age at which ID is first recorded has been reported to peak between 4 and 7 [10]. It was not possible to conduct such an analysis in this study because of insufficient follow-up time. We have, therefore, been cautious in interpreting the results, considering the potential impact of changes in the age at first diagnosis over time. We recommend that future research examines time trends in the prevalence of ID at older ages, including the role of changes in the age at diagnosis, to improve understanding of the underlying mechanisms behind the observed time trend in our study. Thirdly, the possibility of selection bias due to exclusion of individuals who lacked data on the covariates cannot be excluded. Especially, we had to exclude 15% of the total population who did not have data on perinatal factors, including all children born abroad. However, we believe that the selection bias is minimal, as the sensitivity analysis including these individuals showed similar results as the main results (Supplementary Table 3).

## Conclusions

We found that the recorded prevalence of ID diagnoses by age 10 has increased between 2011 and 2021 in Sweden, especially in later years. Associated sociodemographic and perinatal factors, such as birth weight, gestational age, and parental age, migration status, and education at the child’s birth, did not appear to explain the changing prevalence. The observed increase may instead be due to changes in diagnostic practices in Sweden over time. Further studies with longer follow-up time are needed in order to improve planning of health, education, and social services.

## Electronic supplementary material

Below is the link to the electronic supplementary material.

Supplementary Material 1

Supplementary Material 2

## Data Availability

The data are not publicly available due to privacy or ethical restrictions.

## Abbreviations

ADHD: Attention-Deficit/Hyperactivity Disorder
DSM: Diagnostic and Statistical Manual of Mental Disorders
ICD: International Classification of Diseases
ID: Intellectual Disability
NPR: National Patient Register
