# Supplementary material for: Changes in the prevalence of intellectual disability among 10-year-old children in Sweden during 2011 through 2021: a total population study
Source: J Neurodev Disord. 2024 Oct 23;16:58. doi: 10.1186/s11689-024-09576-3 (PMC11515497; doi:10.1186/s11689-024-09576-3)
Supplement: Supplementary file 1 — Supplementary Material 1 [file 11689_2024_9576_MOESM1_ESM.docx]

**Supplementary Figures**

| **ID with autism** | **ID without autism** |
| --- | --- |
|  |  |
| **ID with ADHD** | **ID without ADHD** |
|  |  |
| **Supplementary Figure 1. Changes in intellectual disability among children aged 10 years depicted as crude and adjusted^a^ prevalence ratios in relation to the baseline year 2011, by comorbidity** | |
| a. Adjusted for birth weight, gestational age, and parental migration status, age, and education at child’s birth.  ID = intellectual disability. ADHD = attention-deficit/hyperactivity disorder. | |

| **Profound** | **Severe** |
| --- | --- |
|  |  |
| **Moderate** | **Mild** |
|  |  |
| **Other/unspecific** | |
|  | |
| **Supplementary Figure 2. Changes in intellectual disability among children aged 10 years depicted as crude and adjusted^a^ prevalence ratios in relation to the baseline year 2011, by severity** | |
| a. Adjusted for birth weight, gestational age, and parental migration status, age, and education at child’s birth. | |

|  |
| --- |
| **Supplementary Figure 3. Prevalence for intellectual disability among children aged 10 years, by severity based on the last diagnosis (%)** |
