# Supplementary material for: Changes in the prevalence of intellectual disability among 10-year-old children in Sweden during 2011 through 2021: a total population study
Source: J Neurodev Disord. 2024 Oct 23;16:58. doi: 10.1186/s11689-024-09576-3 (PMC11515497; doi:10.1186/s11689-024-09576-3)
Supplement: Supplementary file 2 — Supplementary Material 2 [file 11689_2024_9576_MOESM2_ESM.docx]

**Supplementary tables:**

| **Supplementary Table 1. Frequency distribution of covariates by birth year** | | | | | | | | | | | | | | | | | | | | | | | | | |
| --- | --- | --- | --- | --- | --- | --- | --- | --- | --- | --- | --- | --- | --- | --- | --- | --- | --- | --- | --- | --- | --- | --- | --- | --- | --- |
|  |  | No. (%) | | | | | | | | | | | | | | | | | | | | | | | |
|  |  | 2001 |  | 2002 |  | 2003 |  | 2004 |  | 2005 |  | 2006 |  | 2007 |  | 2008 |  | 2009 |  | 2010 |  | 2011 |  | Total |  |
| Maternal age at the child's birth (years) | | | | | | | | | | | | | | | | | | | | | | | | | |
|  | <25 | 10,965 | (12.6) | 11,278 | (12.4) | 11,233 | (11.9) | 11,069 | (11.4) | 11,227 | (11.6) | 11,637 | (11.5) | 12,358 | (12.1) | 13,161 | (12.6) | 13,591 | (12.9) | 14,023 | (12.7) | 13,635 | (12.8) | 134,177 | (12.2) |
|  | 25–29 | 27,205 | (31.2) | 27,681 | (30.4) | 27,451 | (29.0) | 27,162 | (27.9) | 26,325 | (27.1) | 27,250 | (27.0) | 27,268 | (26.7) | 28,202 | (27.1) | 28,416 | (27.0) | 29,956 | (27.2) | 29,221 | (27.4) | 306,137 | (27.9) |
|  | 30–34 | 30,860 | (35.4) | 32,980 | (36.2) | 35,068 | (37.0) | 36,938 | (38.0) | 36,919 | (38.0) | 38,023 | (37.6) | 37,297 | (36.5) | 37,101 | (35.6) | 36,698 | (34.8) | 38,333 | (34.7) | 36,631 | (34.4) | 396,848 | (36.2) |
|  | ≥35 | 18,119 | (20.8) | 19,237 | (21.1) | 20,955 | (22.1) | 22,035 | (22.7) | 22,624 | (23.3) | 24,123 | (23.9) | 25,128 | (24.6) | 25,684 | (24.7) | 26,606 | (25.3) | 28,020 | (25.4) | 27,107 | (25.4) | 259,638 | (23.7) |
| Paternal age at the child's birth (years) | | | | | | | | | | | | | | | | | | | | | | | | | |
|  | <25 | 4,861 | (5.6) | 4,888 | (5.4) | 4,996 | (5.3) | 4,737 | (4.9) | 4,806 | (4.9) | 5,041 | (5.0) | 5,449 | (5.3) | 5,922 | (5.7) | 6,099 | (5.8) | 6,229 | (5.6) | 6,101 | (5.7) | 59,129 | (5.4) |
|  | 25–29 | 19,444 | (22.3) | 19,593 | (21.5) | 19,184 | (20.3) | 18,912 | (19.5) | 18,383 | (18.9) | 18,840 | (18.6) | 19,285 | (18.9) | 19,918 | (19.1) | 20,153 | (19.1) | 21,146 | (19.2) | 20,831 | (19.5) | 215,689 | (19.7) |
|  | 30–34 | 30,595 | (35.1) | 32,313 | (35.4) | 33,774 | (35.7) | 35,006 | (36.0) | 35,216 | (36.3) | 35,925 | (35.6) | 34,932 | (34.2) | 34,960 | (33.6) | 34,486 | (32.7) | 35,859 | (32.5) | 34,088 | (32.0) | 377,154 | (34.4) |
|  | 35–39 | 20,914 | (24.0) | 22,417 | (24.6) | 23,919 | (25.3) | 24,362 | (25.1) | 24,230 | (25.0) | 25,737 | (25.5) | 26,060 | (25.5) | 26,631 | (25.6) | 27,494 | (26.1) | 28,866 | (26.2) | 27,563 | (25.9) | 278,193 | (25.4) |
|  | ≥40 | 11,335 | (13.0) | 11,965 | (13.1) | 12,834 | (13.6) | 14,187 | (14.6) | 14,460 | (14.9) | 15,490 | (15.3) | 16,325 | (16.0) | 16,717 | (16.1) | 17,079 | (16.2) | 18,232 | (16.5) | 18,011 | (16.9) | 166,635 | (15.2) |
| Parental education at the child's birth (years) | | | | | | | | | | | | | | | | | | | | | | | | | |
|  | <10 | 3,951 | (4.5) | 3,838 | (4.2) | 3,952 | (4.2) | 3,985 | (4.1) | 4,162 | (4.3) | 4,370 | (4.3) | 4,516 | (4.4) | 4,828 | (4.6) | 4,967 | (4.7) | 5,456 | (4.9) | 5,266 | (4.9) | 49,291 | (4.5) |
|  | 10–12 | 40,761 | (46.8) | 41,544 | (45.6) | 40,870 | (43.2) | 39,965 | (41.1) | 38,882 | (40.0) | 39,296 | (38.9) | 38,730 | (38.0) | 38,363 | (36.8) | 37,815 | (35.9) | 38,649 | (35.0) | 37,197 | (34.9) | 432,072 | (39.4) |
|  | ≥13 | 42,437 | (48.7) | 45,794 | (50.2) | 49,885 | (52.7) | 53,254 | (54.8) | 54,051 | (55.7) | 57,367 | (56.8) | 58,805 | (57.6) | 60,957 | (58.5) | 62,529 | (59.4) | 66,227 | (60.0) | 64,131 | (60.2) | 615,437 | (56.1) |
| Parental migration status | | | | | | | | | | | | | | | | | | | | | | | | | |
|  | Both native-born parents | 67,029 | (76.9) | 70,013 | (76.8) | 72,359 | (76.4) | 73,718 | (75.8) | 72,688 | (74.9) | 75,126 | (74.4) | 74,513 | (73.0) | 75,128 | (72.1) | 75,082 | (71.3) | 77,832 | (70.5) | 74,378 | (69.8) | 807,866 | (73.7) |
|  | Both foreign-born parents | 9,994 | (11.5) | 10,298 | (11.3) | 10,969 | (11.6) | 11,682 | (12.0) | 12,036 | (12.4) | 13,080 | (12.9) | 14,245 | (14.0) | 15,129 | (14.5) | 16,068 | (15.3) | 17,571 | (15.9) | 17,646 | (16.6) | 148,718 | (13.6) |
|  | Foreign-born mother and native-born father | 4,742 | (5.4) | 5,154 | (5.7) | 5,364 | (5.7) | 5,809 | (6.0) | 5,943 | (6.1) | 6,287 | (6.2) | 6,517 | (6.4) | 6,701 | (6.4) | 6,784 | (6.4) | 7,306 | (6.6) | 7,061 | (6.6) | 67,668 | (6.2) |
|  | Native-born mother and foreign-born father | 5,384 | (6.2) | 5,711 | (6.3) | 6,015 | (6.4) | 5,995 | (6.2) | 6,428 | (6.6) | 6,540 | (6.5) | 6,776 | (6.6) | 7,190 | (6.9) | 7,377 | (7.0) | 7,623 | (6.9) | 7,509 | (7.0) | 72,548 | (6.6) |
| Gestational age (week) | | | | | | | | | | | | | | | | | | | | | | | | | |
|  | <28 | 183 | (0.2) | 194 | (0.2) | 209 | (0.2) | 210 | (0.2) | 181 | (0.2) | 222 | (0.2) | 247 | (0.2) | 247 | (0.2) | 269 | (0.3) | 265 | (0.2) | 219 | (0.2) | 2,446 | (0.2) |
|  | 28–31 | 518 | (0.6) | 562 | (0.6) | 548 | (0.6) | 567 | (0.6) | 545 | (0.6) | 571 | (0.6) | 636 | (0.6) | 634 | (0.6) | 592 | (0.6) | 568 | (0.5) | 623 | (0.6) | 6,364 | (0.6) |
|  | 32-36 | 4,435 | (5.1) | 4,738 | (5.2) | 4,841 | (5.1) | 5,087 | (5.2) | 4,858 | (5.0) | 5,120 | (5.1) | 5,015 | (4.9) | 5,162 | (5.0) | 5,211 | (4.9) | 5,462 | (5.0) | 5,275 | (4.9) | 55,204 | (5.0) |
|  | 37-41 | 75,199 | (86.3) | 78,789 | (86.4) | 82,011 | (86.6) | 84,412 | (86.8) | 84,930 | (87.5) | 88,422 | (87.5) | 89,584 | (87.8) | 91,185 | (87.6) | 92,155 | (87.5) | 96,805 | (87.7) | 93,795 | (88.0) | 957,287 | (87.3) |
|  | ≥42 | 6,814 | (7.8) | 6,893 | (7.6) | 7,098 | (7.5) | 6,928 | (7.1) | 6,581 | (6.8) | 6,698 | (6.6) | 6,569 | (6.4) | 6,920 | (6.6) | 7,084 | (6.7) | 7,232 | (6.6) | 6,682 | (6.3) | 75,499 | (6.9) |
| Birth weight (g) | |  |  |  |  |  |  |  |  |  |  |  |  |  |  |  |  |  |  |  |  |  |  |  |  |
|  | <1000 | 192 | (0.2) | 200 | (0.2) | 209 | (0.2) | 200 | (0.2) | 191 | (0.2) | 211 | (0.2) | 260 | (0.3) | 241 | (0.2) | 261 | (0.2) | 262 | (0.2) | 234 | (0.2) | 2,461 | (0.2) |
|  | 1000-1499 | 381 | (0.4) | 390 | (0.4) | 380 | (0.4) | 425 | (0.4) | 396 | (0.4) | 423 | (0.4) | 442 | (0.4) | 445 | (0.4) | 408 | (0.4) | 458 | (0.4) | 427 | (0.4) | 4,575 | (0.4) |
|  | 1500-2499 | 2,911 | (3.3) | 3,151 | (3.5) | 3,232 | (3.4) | 3,223 | (3.3) | 3,227 | (3.3) | 3,540 | (3.5) | 3,417 | (3.3) | 3,638 | (3.5) | 3,588 | (3.4) | 3,686 | (3.3) | 3,615 | (3.4) | 37,228 | (3.4) |
|  | 2500-4499 | 79,888 | (91.7) | 83,537 | (91.6) | 86,980 | (91.8) | 89,377 | (91.9) | 89,649 | (92.3) | 93,185 | (92.2) | 94,477 | (92.6) | 96,168 | (92.3) | 97,167 | (92.3) | 101,865 | (92.3) | 98,461 | (92.4) | 1,010,754 | (92.2) |
|  | ≥4500 | 3,777 | (4.3) | 3,898 | (4.3) | 3,906 | (4.1) | 3,979 | (4.1) | 3,632 | (3.7) | 3,674 | (3.6) | 3,455 | (3.4) | 3,656 | (3.5) | 3,887 | (3.7) | 4,061 | (3.7) | 3,857 | (3.6) | 41,782 | (3.8) |
| Total | | 87,149 | (7.9) | 91,176 | (8.3) | 94,707 | (8.6) | 97,204 | (8.9) | 97,095 | (8.9) | 101,033 | (9.2) | 102,051 | (9.3) | 104,148 | (9.5) | 105,311 | (9.6) | 110,332 | (10.1) | 106,594 | (9.7) | 1,096,800 | (100) |

| Supplementary Table 2. Changes in intellectual disability among children aged 10 years depicted as prevalence ratios in relation to the baseline year 2011 | | | | | | | | | | |
| --- | --- | --- | --- | --- | --- | --- | --- | --- | --- | --- |
| Calendar year | Counts number at risk | Counts cases | Crude | Adjusted for maternal age^a^ | Adjusted for paternal age^a^ | Adjusted for parental migration status^a^ | Adjusted for parental education^a^ | Adjusted for gestational age^a^ | Adjusted for birth weight^a^ | Adjusted for all covariates^b^ |
| 2011 | 87,149 | 555 | 1.00 | 1.00 | 1.00 | 1.00 | 1.00 | 1.00 | 1.00 | 1.00 |
| 2012 | 91,176 | 677 | 1.17 (1.04-1.31) | 1.17 (1.04-1.31) | 1.17 (1.04-1.31) | 1.17 (1.04-1.31) | 1.18 (1.05-1.32) | 1.16 (1.04-1.30) | 1.16 (1.04-1.30) | 1.18 (1.05-1.32) |
| 2013 | 94,707 | 662 | 1.10 (0.98-1.23) | 1.10 (0.98-1.23) | 1.10 (0.98-1.23) | 1.10 (0.98-1.23) | 1.13 (1.01-1.26) | 1.10 (0.98-1.23) | 1.10 (0.98-1.23) | 1.12 (1.00-1.25) |
| 2014 | 97,204 | 643 | 1.04 (0.93-1.16) | 1.04 (0.93-1.17) | 1.04 (0.92-1.16) | 1.03 (0.92-1.16) | 1.08 (0.96-1.21) | 1.04 (0.93-1.16) | 1.04 (0.93-1.17) | 1.06 (0.95-1.19) |
| 2015 | 97,095 | 679 | 1.10 (0.98-1.23) | 1.10 (0.98-1.23) | 1.09 (0.98-1.22) | 1.09 (0.97-1.22) | 1.14 (1.02-1.28) | 1.10 (0.99-1.24) | 1.10 (0.98-1.23) | 1.12 (1.00-1.25) |
| 2016 | 101,033 | 716 | 1.11 (1.00-1.24) | 1.11 (1.00-1.24) | 1.11 (0.99-1.24) | 1.10 (0.98-1.23) | 1.16 (1.04-1.30) | 1.11 (1.00-1.24) | 1.11 (0.99-1.24) | 1.13 (1.01-1.27) |
| 2017 | 102,051 | 768 | 1.18 (1.06-1.32) | 1.18 (1.05-1.31) | 1.17 (1.05-1.30) | 1.15 (1.03-1.29) | 1.24 (1.11-1.38) | 1.18 (1.06-1.32) | 1.18 (1.05-1.31) | 1.19 (1.07-1.33) |
| 2018 | 104,148 | 897 | 1.35 (1.22-1.51) | 1.34 (1.21-1.49) | 1.33 (1.20-1.48) | 1.31 (1.18-1.46) | 1.42 (1.28-1.58) | 1.35 (1.21-1.50) | 1.35 (1.21-1.50) | 1.36 (1.23-1.52) |
| 2019 | 105,311 | 862 | 1.29 (1.15-1.43) | 1.27 (1.14-1.42) | 1.26 (1.14-1.41) | 1.24 (1.11-1.38) | 1.36 (1.22-1.51) | 1.28 (1.15-1.43) | 1.28 (1.15-1.43) | 1.29 (1.16-1.44) |
| 2020 | 110,332 | 1,056 | 1.50 (1.36-1.67) | 1.49 (1.34-1.65) | 1.48 (1.33-1.64) | 1.44 (1.29-1.59) | 1.59 (1.43-1.76) | 1.51 (1.36-1.67) | 1.50 (1.35-1.67) | 1.51 (1.36-1.67) |
| 2021 | 106,594 | 1,062 | 1.56 (1.41-1.74) | 1.55 (1.40-1.72) | 1.53 (1.38-1.70) | 1.49 (1.34-1.65) | 1.65 (1.49-1.84) | 1.57 (1.41-1.74) | 1.56 (1.41-1.73) | 1.57 (1.41-1.74) |
| 2011-2021^c^ | 1,096,800 | 8,577 | 1.04 (1.04-1.05) | 1.04 (1.03-1.05) | 1.04 (1.03-1.05) | 1.04 (1.03-1.04) | 1.05 (1.04-1.06) | 1.04 (1.04-1.05) | 1.04 (1.04-1.05) | 1.04 (1.03-1.05) |
| 1. Adjusted for each covariate 2. Adjusted for parental age, migration status, and education at child’s birth and child’s gestational age and birth weight 3. Average annual relative increase from 2011 to 2021 for the crude and adjusted models | | | | | | | | | | |

| Supplementary Table 3. Changes in intellectual disability among children aged 10 years depicted as prevalence ratios in relation to the baseline year 2011, including those born abroad | | | | | | | |
| --- | --- | --- | --- | --- | --- | --- | --- |
| Calendar year | Counts number at risk | Counts cases | Crude | Adjusted for maternal age^a^ | Adjusted for paternal age^a^ | Adjusted for parental migration status^a^ | Adjusted for all covariates^b^ |
| 2011 | 94,056 | 618 | 1.00 | 1.00 | 1.00 | 1.00 | 1.00 |
| 2012 | 98,910 | 752 | 1.16 (1.04-1.29) | 1.16 (1.04-1.29) | 1.16 (1.04-1.29) | 1.16 (1.04-1.29) | 1.16 (1.04-1.29) |
| 2013 | 102,251 | 753 | 1.12 (1.01-1.25) | 1.12 (1.01-1.25) | 1.12 (1.01-1.25) | 1.12 (1.00-1.24) | 1.12 (1.00-1.24) |
| 2014 | 104,648 | 726 | 1.06 (0.95-1.18) | 1.06 (0.95-1.18) | 1.05 (0.95-1.17) | 1.04 (0.94-1.16) | 1.04 (0.94-1.16) |
| 2015 | 105,191 | 780 | 1.13 (1.02-1.26) | 1.13 (1.02-1.26) | 1.12 (1.01-1.25) | 1.11 (1.00-1.23) | 1.11 (0.99-1.23) |
| 2016 | 110,268 | 807 | 1.11 (1.00-1.24) | 1.11 (1.00-1.24) | 1.11 (1.00-1.23) | 1.09 (0.98-1.21) | 1.08 (0.97-1.20) |
| 2017 | 112,077 | 860 | 1.17 (1.05-1.30) | 1.16 (1.05-1.29) | 1.15 (1.04-1.28) | 1.13 (1.02-1.25) | 1.12 (1.01-1.24) |
| 2018 | 114,414 | 1,004 | 1.34 (1.21-1.48) | 1.33 (1.20-1.47) | 1.32 (1.19-1.46) | 1.28 (1.16-1.41) | 1.26 (1.14-1.40) |
| 2019 | 117,414 | 1,000 | 1.30 (1.17-1.43) | 1.28 (1.16-1.42) | 1.28 (1.15-1.41) | 1.23 (1.11-1.36) | 1.22 (1.10-1.35) |
| 2020 | 122,460 | 1,227 | 1.53 (1.38-1.68) | 1.51 (1.37-1.66) | 1.50 (1.36-1.65) | 1.43 (1.30-1.58) | 1.42 (1.28-1.56) |
| 2021 | 118,761 | 1,235 | 1.58 (1.44-1.74) | 1.57 (1.42-1.73) | 1.55 (1.41-1.71) | 1.47 (1.34-1.62) | 1.46 (1.32-1.60) |
| 2011-2021^c^ | 1,200,450 | 9,762 | 1.04 (1.04-1.05) | 1.04 (1.03-1.05) | 1.04 (1.03-1.05) | 1.04 (1.03-1.04) | 1.03 (1.03-1.04) |
| 1. Adjusted for each covariate 2. Adjusted for parental age and migration status 3. Average annual relative increase from 2011 to 2021 for the crude and adjusted models | | | | | | | |
